# Lipid production and cellular changes in *Fremyella diplosiphon* exposed to nanoscale zerovalent iron nanoparticles and ampicillin

**DOI:** 10.1186/s12934-023-02113-2

**Published:** 2023-06-07

**Authors:** Yavuz S. Yalcin, Busra Aydin, Huan Chen, Samson Gichuki, Viji Sitther

**Affiliations:** 1 Department of Biology, Morgan State University, Baltimore, MD 21251 USA; 2 National High Magnetic Field Laboratory, Ion Cyclotron Resonance Facility, Florida State University, 1800 East Paul Dirac Dr, Tallahassee, FL 32310-4005 USA

**Keywords:** Antibiotic, Cyanobacteria, Fatty acids, Fluorescence, Nanoparticles, Transmission Electron Microscopy

## Abstract

With the dramatic decrease in fossil fuel stocks and their detrimental effects on the environment, renewable energy sources have gained imminent importance in the mitigation of emissions. As lipid-enriched energy stocks, cyanobacteria are the leading group of microorganisms contributing to the advent of a new energy era. In the present study, the impact of Nanofer 25 s nanoscale zero-valent iron nanoparticles (nZVIs) and ampicillin on lipid production and cellular structural changes in *Fremyella diplosiphon* strain B481-SD were investigated. Total lipid abundance, fatty acid methyl ester (FAME) compositions, and alkene production as detected by high-resolution two-dimensional gas chromatography with time-of-flight mass spectrometry (GC × GC/TOF–MS) was significantly higher (*p* < 0.05) in the individual application of 0.8 mg/L ampicillin, 3.2 mg/L nZVIs, and a combined regimen of 0.8 mg/L ampicillin and 3.2 mg/L nZVIs compared to the untreated control. In addition, we identified significant increases (*p* < 0.05) in monounsaturated fatty acids (MUFAs) in *F. diplosiphon* treated with the combination regimen compared to the untreated control, 0.8 mg/L of ampicillin, and 3.2 mg/L of nZVIs. Furthermore, individual treatment with 0.8 mg/L ampicillin and the combination regimen (0.8 mg/L ampicillin + 3.2 mg/L nZVIs) significantly increased (*p* < 0.05) Nile red fluorescence compared to the untreated control, indicating neutral membrane lipids to be the main target of ampicillin added treatments. Transmission electron microscopy studies revealed the presence of single-layered thylakoid membranes in the untreated control, while complex stacked membranes of 5–8 layers were visualized in ampicillin and nZVI-treated *F. diplosiphon*. Our results indicate that nZVIs in combination with ampicillin significantly enhanced total lipids, essential FAMEs, and alkenes in *F. diplosiphon*. These findings offer a promising approach to augment the potential of using the strain as a large-scale biofuel agent.

## Introduction

World oil consumption has increased tremendously from 64 to 100.3 million barrels per day between 1990 and 2021 due to rising population and economic growth, with a projected rise of 40–50% by 2030 [1]. Annual oil consumption accounts for 1 in 46 of the total reserves, which would be insufficient to meet this exorbitant demand. Furthermore, the ever-increasing demand for fossil fuels has resulted in detrimental environmental effects; for example, carbon emissions have increased by 4.4% despite being limited to the maximum anthropogenic warming of 1.5 °C within 10 years [2]. Consequently, sustainable and indigenous renewable energy sources have gained immense significance as clean green technology and are widely embraced by consumers and corporations [3]. Thus, protecting the planet Earth from the inevitable consequences of burning fossil fuels could be achieved by integrating alternative bioenergy sources [4]. The emerging use of cyanobacteria as third and fourth-generation energy sources has led these vital cell factories to be extensively explored for alternative energy [5].

Cyanobacteria offer a promising platform for biofuel production due to their readily available genome sequences, easy manipulation of genetic material, and the ability to thrive in marginal areas with minimal nutrient requirements. In cyanobacteria, lipids constitute approximately 20% of dry mass weight under standard growth conditions; however, external factors are known to trigger lipid synthesis by 30–68% [4]. This might be reasoned by a well-known phenomenon, the hormetic effect, which results in increased cellular growth and pigmentation when exposed to external factors such as metallic nanoparticles and antibiotics. Thus, these organisms have found applications as an industrialized lipid source for third-generation biofuel production. However, it should be noted that the application of cyanobacterial lipids for biofuel production should not only be regarded in terms of lipid quantity but also the quality of extracted lipids. Hence, it is crucial to achieve breakthroughs in metabolic engineering to increase lipid yield per harvest volume, thus reducing extraction costs per unit product to unlock their potential as cost-effective biofuel alternatives.

Both neutral and polar lipids from cyanobacteria are converted to biofuel through various processes, such as hydrothermal liquefaction and transesterification, and used as indicators of potential biofuel-yielding strains. In biofuel production, lipid quality and diversity are determined by carbon number distribution, fatty acid chain length, and the number of double bonds [6–8]. As an indicator of biofuel quality, the presence of carbon chain lengths of C_10_ or longer is considered more valuable for biodiesel properties [9]. For example, 50% of the fatty acids in the filamentous cyanobacterium, *Trichodesmium erythraeum,* were found to consist of C_10_-C_12_ carbon chains [10]. Additionally, *Phormidium* and *Prochlorothrix* species have been reported to have C_14_ carbon chains accounting for up to 30% of total lipids, the major type of fatty acid methyl esters (FAMEs) [11].

Cyanobacterial FAMEs contribute significantly to the development of high-quality biodiesel. Biodiesel quality is related to the composition of FAMEs, in which cetane numbers, ignition temperature, viscosity, and oxidative stability play a significant role [12]. For example, a fuel mixture consisting of high saturated FAMEs with high cetane numbers will result in delayed fuel combustion time and smooth burning. Higher resistance during the oxidation of biofuels due to a large number of saturated bonds and glycerol polymerization prevents combustion from occurring, thus significantly increasing the reliability of engines [13].

Solvent-based techniques such as GC × GC/TOF–MS and gravimetric methods are well-known methods for detecting total lipid abundance and FAME distribution in cyanobacteria [14]. However, fluorescence-based methods such as Nile red and Boron-Dipyrromethene (BODIPY) staining probes have several advantages but have not been adequately studied [15]. These techniques have become prominent in quantifying lipids owing to the rapid detection of intracellular lipid distribution and are easily applicable to various cyanobacterial strains. The aforesaid advantages of fluorescence-based techniques, along with solvent-based methods, have the potential to provide much more detailed and precise results. However, there are no studies on the combined effects of nanoscale zero-valent iron nanoparticles (nZVIs) and ampicillin on fatty acid production and cellular changes in *Fremyella diplosiphon*, a model cyanobacterium. We report enhanced lipid productivity and related intracellular membrane changes in *F. diplosiphon* exposed to optimal nZVIs and ampicillin concentrations.

## Materials and methods

### Growth conditions, zero-valent iron nanoparticles, and ampicillin

*Fremyella diplosiphon* B481-SD strain, overexpressed with the sterol desaturase gene (accession MH329183), was used in this study. Active cultures were grown in Blue-Green-11 (BG-11) medium and 4,2-Hydroxyethyl, 1-piperazineethanesulfonic acid (HEPES) under wide-spectrum red light (650 nm) and shaking continuously at 170 rpm at 28 °C in an Innova 44R shaker (Eppendorf, Hamburg, Germany) [16]. The light fluence rate was adjusted to 30 µmol/m^2^/s using the model LI-190SA quantum sensor (Li-Cor, USA). These conditions were kept constant during the study.

Zero-valent iron nanoparticles used in this study were obtained from Nano Iron company (Rajhrad, Czech Republic). These nZVIs have been previously characterized in our laboratory, and the average particle size determined to be approximately 53 nm [17]. Ampicillin, one of the β-lactam group antibiotics targeting cellular membranes, was used in this study. An optimal concentration of 0.8 mg/L ampicillin and 3.2 mg/L of nZVIs was selected based on previous reports by Yalcin et al. [18] and Fathabad et al. [19] for *F. diplosiphon.* B481-SD cultures at 0.6 OD_750_ were treated with ampicillin (0.8 mg/L), nZVIs (3.2 mg/L), and the combined regimen of both ampicillin (0.8 mg/L) and nZVIs (3.2 mg/L). Control cultures were grown in the absence of antibiotics or nZVIs. Three replicated treatments were maintained and the experiment repeated once.

### Total lipid extraction and GC × GC/TOF–MS analysis of transesterified lipids in *F. diplosiphon* treated with optimal nZVIs and ampicillin

To eliminate organic and inorganic contaminants, B481-SD cultures were grown in flasks cleaned by heating for 4 h at 500 °C in a muffle oven (Thermo Fisher Scientific, MA, USA). B481-SD cultures grown under conditions mentioned in 2.2 were harvested on day 15. Total lipids were extracted using the chloroform: methanol method described by Folch et al. [20]. Briefly, cultures were pelleted and lyophilized, and 15 mL methanol/chloroform (2:1, v/v) solvent was added to 100 mg dry weight of cells. The mixture was homogenized, incubated on ice, and centrifuged at 3000 rpm for 10 min to collect the organic phase. The biomass residue was re-extracted thrice with 4 mL of methanol and 2 mL of chloroform. The lower organic phase was dried in a rotary evaporator (Heidolph, IL, USA), and the total lipid content was estimated by gravimetry.

Simultaneous extraction and transesterification of *F. diplosiphon* lipids were performed according to the method described by Wahlen et al. [21]. B481-SD cells grown in 3.2 mg/L nZVIs, 0.8 mg/L ampicillin, and the combination regimen were lyophilized (100 mg), dissolved in 3 mL methanol containing 1.8% (v/v) sulfuric acid, and exposed to 80 °C for 20 min in a commercial multimode scientific microwave (CEM Corporation, USA) with a maximum power output set at 25W per sample. The reaction was quenched with 4 ml chloroform, washed in distilled water, and centrifuged at 2000 rpm for phase separation. The chloroform phase containing FAMEs and lipids was transferred to a new flask, and the remaining biomass was washed twice with 2 mL chloroform and mixed. The extracted FAMEs were subjected to high-resolution two-dimensional gas chromatography-time of flight mass spectrometry (GC × GC/TOF-MS) as previously described by Tabatabai et al. [6].

### BODIPY staining

To stain glycolipids in neutral lipids and the layered membrane surrounding the lipid droplets, boron-dipyrromethene difluoride (BODIPY) 493/503 (Molecular Probes, Thermo Fisher Scientific, Waltham, MA, USA) was used. B481-SD cultures were grown to an optical density of 0.6 (OD_750_) under conditions mentioned 2.1. One ml of cells was collected by centrifuging at 4000 rpm, resuspended in 1 ml phosphate-buffered saline (PBS) at pH 7.5, and incubated for 1 h at room temperature. Next, the cells were centrifuged, and the pellet was washed in 0.5 ml PBS (pH 7.5). The washed cells were mixed with 1 μl of 50 ng/ml BODIPY in dimethyl sulfoxide (DMSO). The cell suspension was incubated at room temperature in the dark for 15 min. After staining, *F. diplosiphon* cultures were placed on the slides covered with 1.5% agarose and observed by a Leica DM 2500 confocal fluorescence microscope. Green fluorescence signal was monitored with a BP470-40-nm excitation filter and a BP525-50-nm emission filter [22].

### Nile Red Staining

The modified Nile Red staining procedure described by Chen et al. [23] was used in this study. A stock solution of 250 mg l:1 in acetone Nile Red (9-diethylamino-5H benzo[alpha]phenoxazine-5-one, Sigma Aldrich., MO, USA) was prepared. B481-SD cultures were grown to an optical density of 0.6 (OD_750_) under conditions mentioned 2.1. Next, 150 μL of cultures suspended in 50 μL of 25% DMSO, and 50 μL of BG-11 as control were pipetted into black-sided clear-bottomed plates (Corning Inc., NY, USA). Nile red concentration was optimized to 1.0 μg/mL and added to the untreated and treated B481-SD cultures. After incubation at 35 °C in the dark for 35 min, fluorescence measurements were made at excitation of 530 nm and emission of 570 nm wavelengths, using a Synergy H1 Multimode microplate reader equipped with Gen5 software. Cells that were not stained with Nile red served as a negative control. In addition, the stain alone was used to assess autofluorescence effects.

### Transmission electron microscopy

For electron microscopy studies, cultures were harvested by centrifugation at 4,000 rpm for 10 min and fixed with glutaraldehyde (2.5% final concentration) for 5 min at room temperature. After washing three times with 5 mM HEPES (pH 8.0), the samples were post-fixed with 2% potassium permanganate solution at 4 °C overnight. This step served to contrast the glycolipid layers better. The cells were then washed 8 to 10 times with ddH_2_O and immersed in 2% Sea Kem agarose (FMC Bioproducts, Rockland, USA). After solidification, the agar blocks were cut into small cubes with an edge length of ~ 2 mm and dehydrated by incubation in increasing alcohol concentrations (v/v) (70%, 80%, 90%, and 95%) two times each for 10 min and then for 20 min with three changes in 100% ethanol. The samples were initially prepared by incubation with propylene oxide for 15 min to infiltrate Epon successfully. Infiltration of the samples with resin was carried out in a 1:2 mixture of propylene oxide and Epon for 30 min, followed by a 1:3 mixture for 90 min at RT. Finally, the agarose cubes were embedded in Epon pure and polymerized for 24 h at 40 °C and for 48 h at 60 °C.

Ultrathin sections were made using a Transmission Electron Microscope (TEM) Philips Tecnai 10 at 80 kV, and micrographs recorded with an exposure time of 2 s in a medium format film and digitized using a transmitted light scanner [24]. This was performed at the Johns Hopkins University microscope facility (Baltimore, MD, USA).

### Statistical analysis

Significance among cumulative treatment means was determined using ANOVA and Tukey's honest significant differences post hoc test at 95% confidence intervals (*p* < 0.05). The single factor, fixed-effect ANOVA model, Yij = μ + αSi + εij, was used where Y is the total lipid content in strain i and biological replicate j. The μ represents overall total lipid content with adjustments from the effects of strain (αS), and εij is the experimental error from strain i and biological replicate j.

## Results and discussion

It is known that cyanobacterial lipid content could be enhanced between 7 and 32% of the biomass composition by manipulating culture conditions like temperature, light, salinity, carbon dioxide concentration, nitrogen starvation, phosphorus deficiency, and exogenous stresses such as ultrasonication, ultraviolet radiation, and TiO2 [25, 26]. In a study by Singh et al. [27] it was reported that although *Leptolyngbya foveolarum* growth was proficient at pH ranging from 6.5 to 9.0, the maximal lipid content was achieved at pH 7.5. As Nile red and BODIPY have different characteristics in their hydrophilic domains and fluorescent emissions, Nile red is preferred for the detection of triacylglycerols (TAGs), but BODIPY for lipid droplets [15]. Therefore, the tendency of these stains to bind to unique lipid subtypes and reflect fluorescence provides crucial data on lipid distribution. Despite the promising advantages of these fluorescence dyes, to our knowledge, these methods have not been tested on *F. diplosiphon* lipids. Thus, in the present study, fluorescent-based lipid detection and quantification in nZVI and ampicillin-treated *F. diplosiphon* was combined with conventional methods.

### Nile Red and BODIPY analysis of B481-SD strain treated with nZVIs and ampicillin

Detecting intracellular lipid distribution in cyanobacteria and microalgae using Nile Red and BODIPY has gained increased attention in recent years [28]. The attachment of each unique fluorescent dye to different groups of lipids, along with their distinctive fluorescence spectra, elicits the detection of intracellular lipids at different emission wavelengths. In the present study, we used spectral light measurement to categorize and analyze the effect of nZVIs and ampicillin on *F. diplosiphon* cells.

**Fig. 1:**
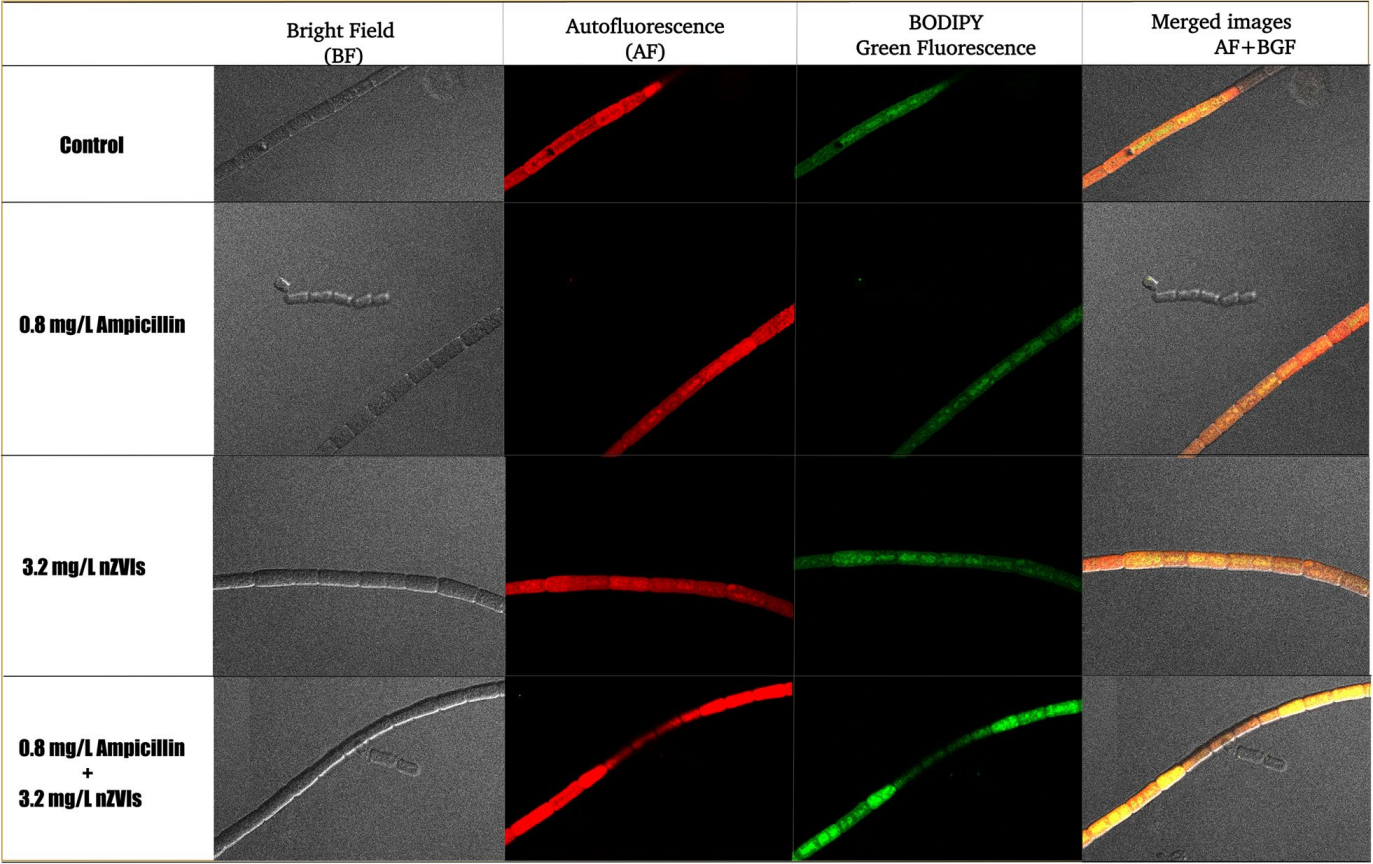
Expression of fluorescent polyhydroxyalkanoate (PHA) bodies in *Fremyella diplosiphon*. Images are respentative of B481-SD untreated control, 0.8 mg/L ampicillin, 3.2 mg/L nZVIs, and the combination regimen of 0.8 mg/L ampicillin + 3.2 mg/L nZVIs in phase contrast image of bright field (BF), autofluorescence (AF), BODIPY green flurorescence (BGF), and merged images (AF + GF)

We observed BODIPY stain round-shaped fluorescent structures in *F. diplosiphon* treated in the combination regimen of 3.2 mg/L nZVIs and 0.8 mg/L ampicillin; however, these structures were reduced in the untreated control (Fig. 1). We reason that the increase in fluorescence activity in BODIPY-stained cells might be attributed to polyhydroxyalkanoate (PHA) inclusion bodies, which are cyanobacterial carbon and energy storage structures covered by phospholipid layers [29]. Our results are corroborated by a report by Hong et al. [30] in which stained granules in various cyanobacterial species, including *Synechocystis sp.* WHSYN, *Nodularia sp.* Las Olas and *Phormidium* cf. *iriguum* CCALA 759 were observed.

**Fig. 2:**
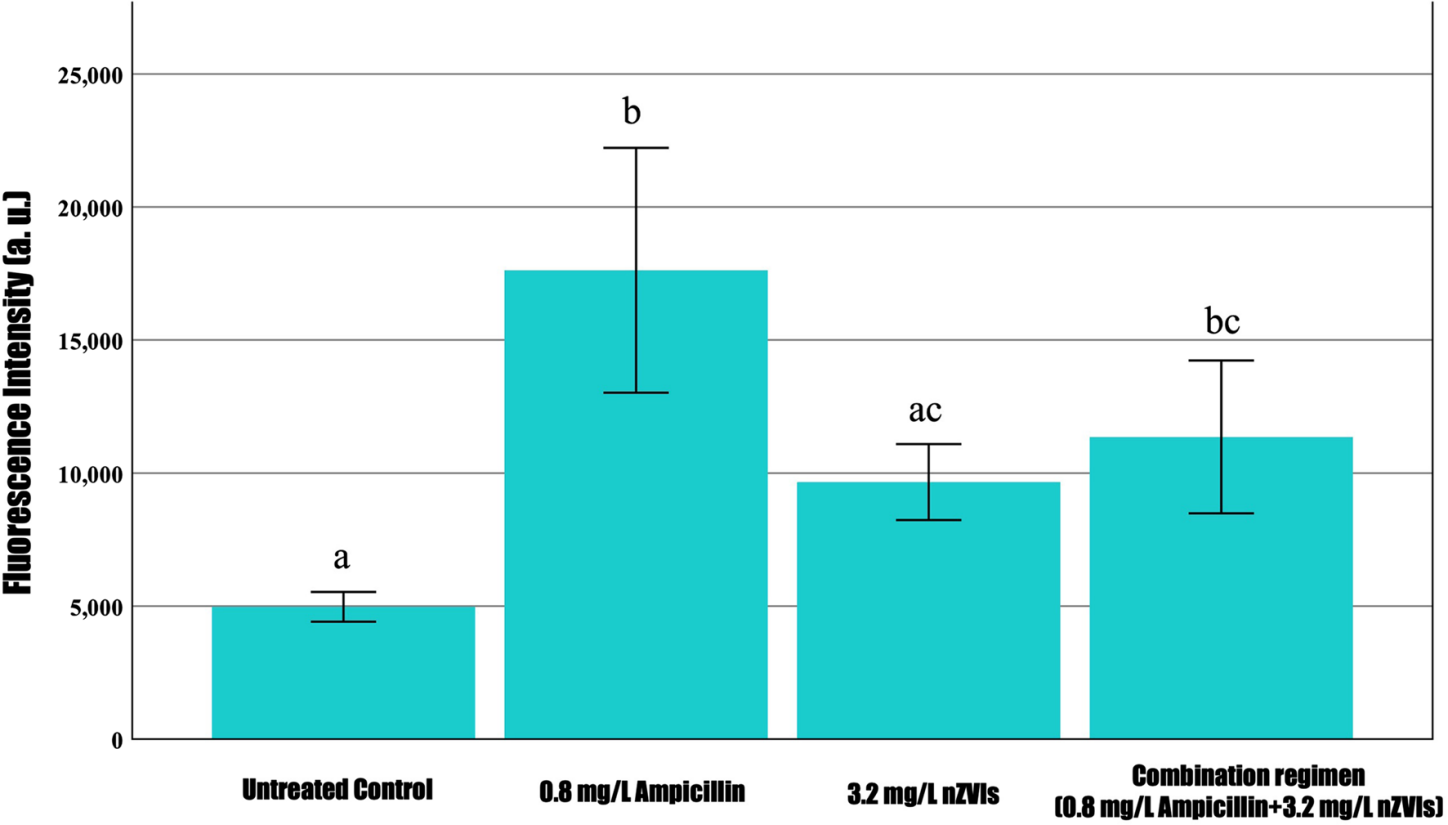
Nile red fluorescence intensity in *Fremyella* *displosiphon* treated with 0.8 mg/L ampicillin, 3.2 mg/L nZVIs, combination regimen (0.8 mg/L ampicillin and 3.2 mg/L nZVIs), and untreated contol at peak excitation and emission wavelengths of 530 nm and 575 nm. The error bars labeled with different letters (e.g., a and ac and bc) are statistically significantly different (*p* < 0.05); however, same letters (e.g., a and ac or ac and bc) indicate no significant difference (*p* > 0.05) among groups (Tukey's post-hoc test). Error bars indicate the standard error (SE) of the mean

To obtain quantitative results via Nile red-associated lipid analysis, fluorescent intensity was measured at 530 nm excitation and 575 nm emission in a microplate reader. Noticeably, a significant increase in Nile red fluorescence was observed in 0.8 mg/L ampicillin-treated cells relative to the untreated control and 3.2 mg/L nZVIs, except in the combination regimen (0.8 mg/L ampicillin and 3.2 mg/L nZVIs) as shown in Fig. 2. Thus, we speculate that ampicillin, which was common in both treatments, could have facilitated *F. diplosiphon* cells to produce neutral lipids. It is known that ampicillin targets β-lactam groups in the cell membrane, which could significantly affect cellular lipid proportions [31]. In a previous study by Yalcin et al. [18], ampicillin at 0.8 mg/L was shown to increase membrane permeability. Thus, abundant lipids induced by the low-dose stimulation effect of ampicillin could have contributed to increased Nile red fluorescent activity. Although Nile red is known to stain TAGs and membrane lipids, it does not stain intracellular droplets, since emission fluorescence shifts the interaction between Nile red and hydrophobic cellular protein compartments [32]. Our observations indicate that the activity of nZVIs is reduced in the absence of ampicillin, thus resulting in decreased Nile red fluorescent activity and lowering the amounts of lipids.

### GC x GC/TOF–MS analysis of transesterified lipids in *F. diplosiphon* treated with optimal nZVIs and ampicillin

**Fig. 3:**
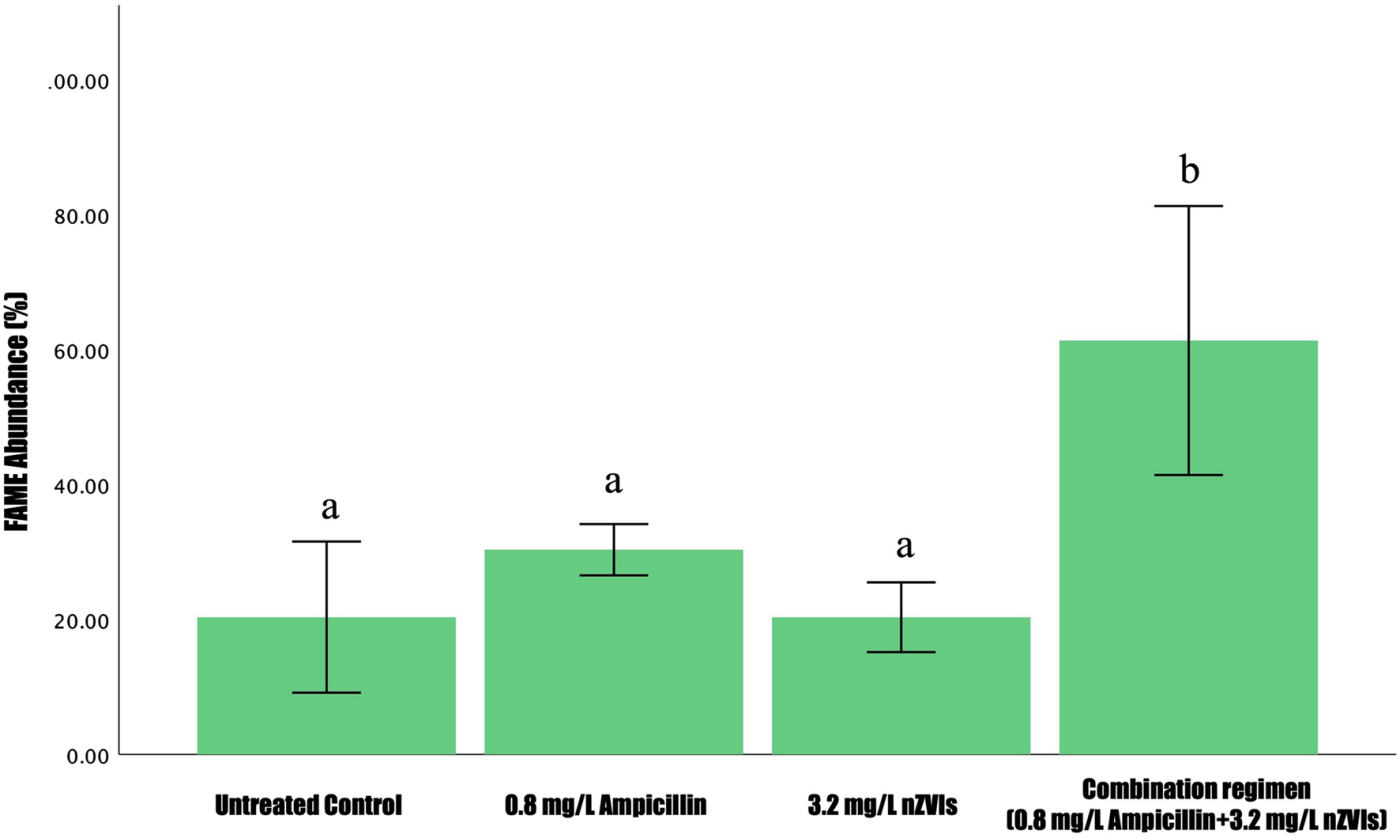
Comparison of fatty acid methy ester (FAME) abundance in *Fremyella displosiphon* treated with 0.8 mg/L ampicillin, 3.2 mg/L nZVIs, combination regimen of 0.8 mg/L ampicillin and 3.2 mg/L nZVIs, and untreated control. Different letters **a** and **b** above the error bars indicate statistical significance (*p* < 0.05) among treatment means (Tukey’s post-hoc test). Error bars indicate the standard error (SE) of the mean

We used conventional methods to quantify lipid subtypes and lipid yield produced in B481-SD. We observed the combination regimen of 0.8 mg/L ampicillin and 3.2 mg/L nZVIs to significantly increase (*p* < 0.05) total lipids compared to the control, 0.8 mg/L ampicillin, and 3.2 mg/L nZVIs (Fig. 3). Although a slight increase in total lipid content was observed in 0.8 mg/L of ampicillin compared to the control and 3.2 mg/L nZVIs, it was not statistically different (*p* > 0.05) from the relative abundance of FAMEs quantified by GC × GC/TOF-MS (Fig. 3). We speculate that the difference in the detection of lipids using Nile-red staining and GC × GC/TOF–MS is possibly due to the different types of lipids detected. While Nile red primarily detects membrane lipids, GC × GC/TOF–MS detects the lipids that can be converted to FAMEs. In addition, nZVIs, which are characterized as zero-valent Fe^0^, are converted Fe^+3^ by cyanobacterial cellular processes involved in photosynthesis and lipid production, and its absorption into intracellular space might be facilitated due to ampicillin-associated increase in membrane permeability [33].

It is well-known that photosynthetic organisms such as cyanobacteria and microalgae are indispensable sources of lipids. Although total lipid abundance has a strong correlation to biofuel production and performance, FAME composition analysis has recently gained more prominence in providing absolute analysis. GC × GC/TOF–MS of transesterified FAMEs in B481-SD grown in the combination regimen of 0.8 mg/L ampicillin and 3.2 mg/L nZVIs revealed the occurrence of hexadecanoic acid, methyl ester (C16:0), ç-Linolenic acid (C18:3), methyl ester, methyl stearidonate (C18:4), 9,12-Octadecadienoic acid (C18:2), methyl ester, 9-Octadecenoic acid, methyl ester (C18:1), 7-hexadecenoic acid, methyl ester (C16:1). These FAMEs have been previously reported by Tabatabai et al. [25] and Fathabad et al. [34] when *F. diplosiphon* cells were subjected to gold and iron nanoparticles, respectively. Accordingly, hexadecenoic acid and methyl ester (C16:0) constituted the main lipid component in our study, and was detected in all treatment groups, including the untreated control (66.4%), 3.2 mg/L (52.6%), and 25.6 mg/L (51.9%).

**Fig. 4:**
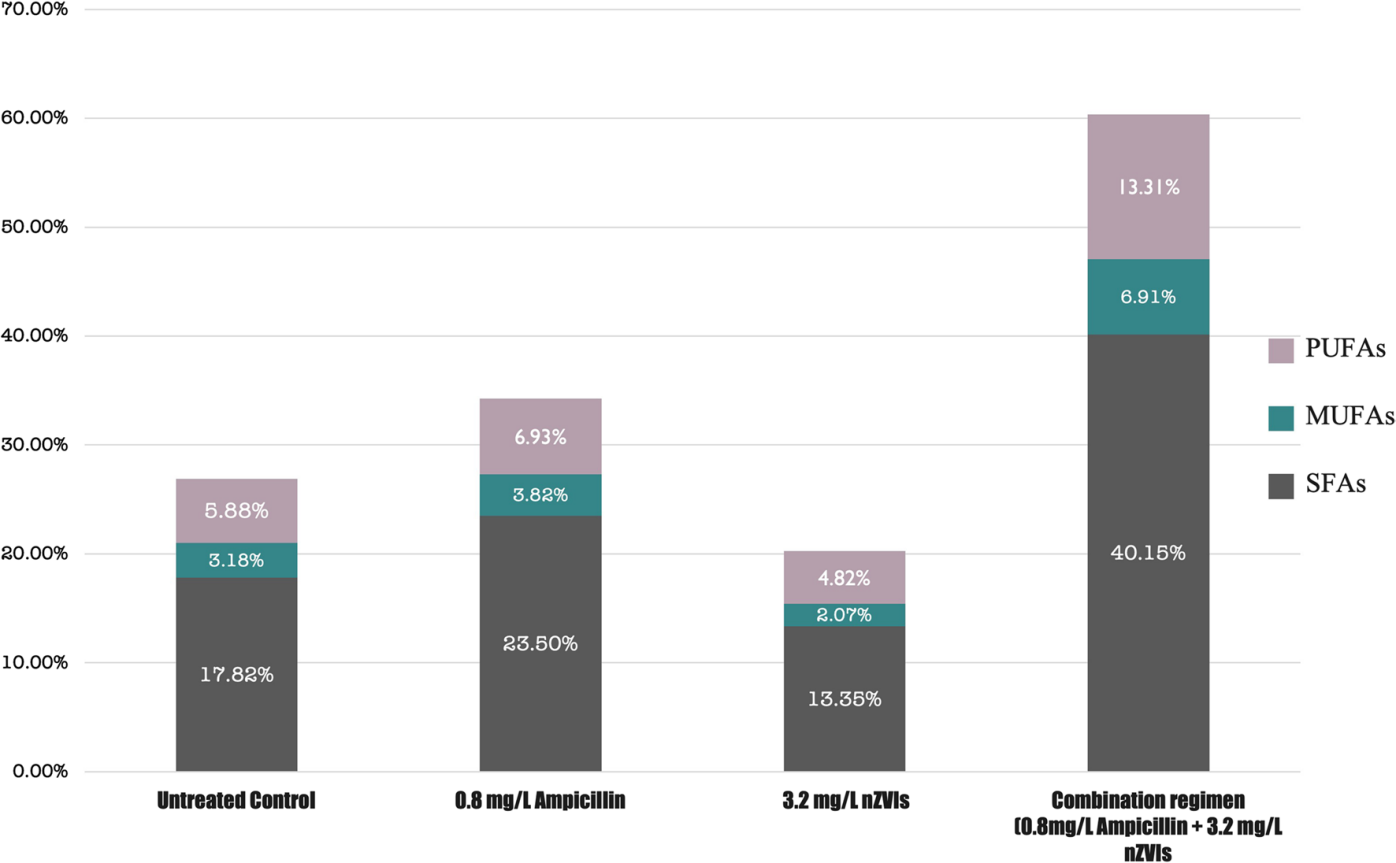
Saturated and unsaturated fatty acid methyl ester (FAME) percentages in the total FAMEs of *Fremyella displosiphon* untreated control and cultures treated with 0.8 mg/L ampicillin, 3.2 mg/L nZVIs, and the combination regimen of 0.8 mg/L ampicillin, 3.2 nZVIs. *SFAs (saturated fatty acids), PUFAs (polyunsaturated fatty acids), MUFAs (monounsaturated fatty acids)

Our results paralleled another report by Fathabad et al. [19] in which C16 was found to be the main FAME component in *F. diplosiphon*. Several studies have reported C16 as the dominant FAME, ranging from 23 to 43% in various cyanobacterial and microalgal species [35–37]. In addition to the fatty acid composition, another crucial criterion for biofuel quality is the quantity of fatty acid saturation. To achieve ideal biodiesel properties, the most preferred group of fatty acids are the monounsaturated fatty acids (MUFAs) [38], followed by polyunsaturated fatty acids, and lastly the saturated fatty acids (SFA). In our study, a 2-3 fold increase in *F. diplosiphon* MUFAs was observed in the combination regimen compared to the untreated control (Fig. 4). Although this remarkable increase is expected due to the augmentation of the total lipids, it was not significant (*p* > 0.05) at 0.8 mg/L ampicillin, and a decrease in MUFAs to SFAs ratio when compared to the control and the combination regimen was observed. Therefore, we hypothesize that the stearoyl-CoA desaturase enzyme, which is an iron-containing molecule, catalyzes the conversion of SFAs to MUFAs [39], and nZVIs might facilitate the activity of this enzyme.

It should be noted that even if cyanobacteria-based biofuels achieve ideal properties, the environment cannot be controlled, which could drastically impact their performance. For instance, lower temperatures could increase oxidative instability, hindering maximal biodiesel power achievement [40]. Thus, it is essential to consider the environmental conditions to achieve the perfect biodiesel blend. Consensus on the high lipid quality provided by MUFAs, it is well established that this type of lipids can be easily degraded under extreme conditions; however, SFAs are not categorized as one of the most yielding types of lipids and preferred in extreme conditions due to their resilience [41]. Therefore, it is possible to provide the best combination of lipids with an amicable ratio between the SFAs and MUFAs, depending on the environmental conditions.

**Fig. 5:**
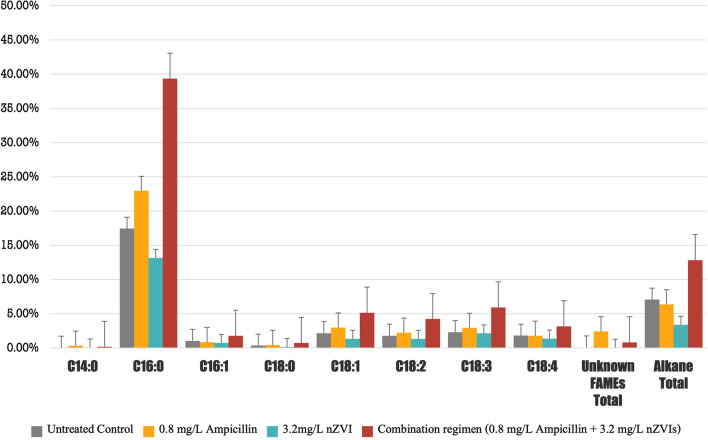
Comparison of fatty acid methyl ester (FAME) composition of *Fremyella diplosiphon* strain B481-SD total lipids subjected to direct transesterification in untreated control, 0.8 mg/L ampicillin, 3.2 mg/L nZVIs, and the combination regimen (0.8 mg/L ampicillin and 3.2 mg/l nZVIs). Average percent FAME (±standard error) for three biological replicates of each strain is shown. Error bars indicate the standard error (SE) of the mean

In the transesterified lipids of B481-SD treated with 3.2 mg/L nZVIs and 0.8 mg/L ampicillin, methyl ester of hexadecanoic acid (C16:0), methyloctadecenoate (C18:1) methyl octadecadienoate (C18:2) and ç-Linolenic acid (C18:3) levels were significantly higher than (*p* < 0.05) the untreated control (Fig. 5). Nevertheless, lipids produced in *F. diplosiphon* cells treated with nZVIs at 3.2 mg/L or ampicillin at 0.8 mg/L were not statistically different (*p* > 0.05) when compared to the untreated control. Therefore, we conclude that a combination regimen could be more effective in increasing FAME compositions and total lipid abundance due to their cumulative effect.

Alkanes are byproducts of lipid metabolism and are primarily produced in the cyanobacterial thylakoid membrane and other photosynthetic organisms as well. Other major types of alkenes in cyanobacteria are pentadecane and heptadecane (C15–C19) structures, which are long-chain groups of alkanes and are most suitable for combustion speed and ignitability [42]. However, cyanobacteria have the added benefit of producing alkanes in their cells, which increases their value for biodiesel production while making it economical [43]. Therefore, enhancing the alkane production per unit via nZVIs and ampicillin treatment in *F. diplosiphon* offers an incentive to improve cyanobacterial biofuel production. In this study, we observed the combination regimen of 0.8 mg/L ampicillin and 3.2 mg/L nZVIs to result in a 2-4-fold (*p* < 0.05) increase in total alkane quantity compared to untreated control, 0.8 mg/L of ampicillin, and 3.2 mg/L of nZVIs (Fig. 5). We hypothesize that an increase in the number of alkanes in the combination regimen might be due to nZVI activity, leading to the augmentation of total lipids produced. Furthermore, a significant increase (*p* < 0.05) in ampicillin-associated total FAMEs was observed in 0.8 mg/L ampicillin alone and the combination regimen (0.8 mg/L ampicillin and 3.2 mg/L nZVIs); however, there were no significant changes in alkane content. Therefore, we reason that iron molecules could have played a critical role as cofactors in the process of photosynthesis, which occurs in the thylakoid membrane containing abundant lipid molecules. Thus, the increased activity in these unique structures could have enhanced alkane production. Alkane biosynthesis could be facilitated by stress factors such as ampicillin, which could have enhanced the cellular intake of metallic nanoparticles such as nZVIs.

### Transmission Electron Microscopy

Transmission Electron Microscopy was used to gain insight into the structural changes in the cell membrane and thylakoids in *F. diplosiphon* treated with nZVIs, ampicillin, and a combined regimen of nZVIs and ampicillin.

#### Cell membrane alterations

**Fig. 6:**
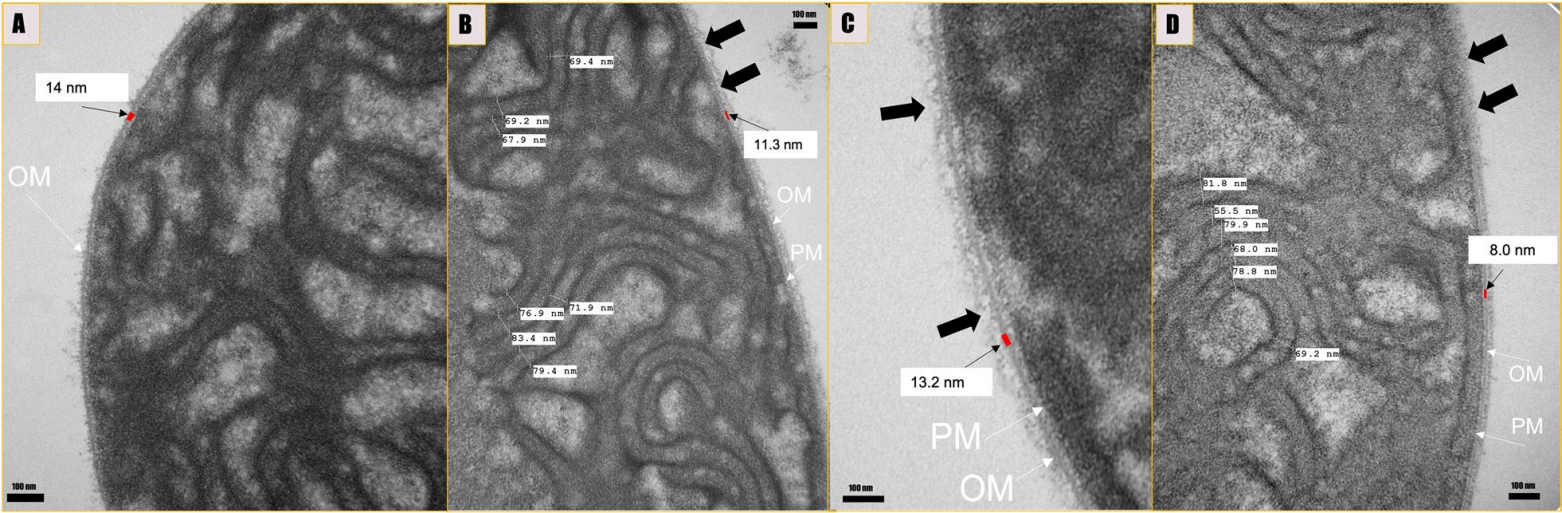
Membrane changes in *Fremyella displosiphon* B481-SD untreated control (**A**), 0.8 mg/L, ampicillin (**B**), 3.2 mg/L nZVIs (**C**) and the combination regimen 0.8 mg/L ampicillin and 3.2 mg/L NZVIs (**D**) observed by transmission electron miscroscopy. Outer membrane discontinuity and thinnng (black arrows) with changes in plama membrane (PM) and outer membrane (OM) were observed. Alterations in the membrane thickness among the treatment groups and untreated controls are indicated by red rectangles

While intact and thickest cellular membrane was observed in the untreated control (Fig. 6A), disruption of membrane integrity and cellular membrane thinning were visualized in 0.8 mg/L ampicillin and the combination regimen of 0.8 mg/L ampicillin and 3.2 mg/L nZVI (Fig. 6B, D). In addition, as reported in our previous study, extracellular stressors such as antibiotics increase membrane permeability as detected by lactate dehydrogenase assay [18]. Despite the alteration of the outer membrane structure (Fig. 6D), we observed that the cells maintained functions and survived in the combination regimen 0.8 mg/L ampicillin and 3.2 mg/L nZVIs. Cyanobacterial filament structures maintain intracellular network, structural durability, and transient connections that permit lipid exchange. In addition, thylakoid membranes provide filament stability and function by attaching to the peripheral cytoplasm and are closely related to cytoplasmic membranes, maintaining the cellular structure to enable cells survive stress conditions.

#### Thylakoid membrane structural adaptations in response to environmental stressors

**Fig. 7:**
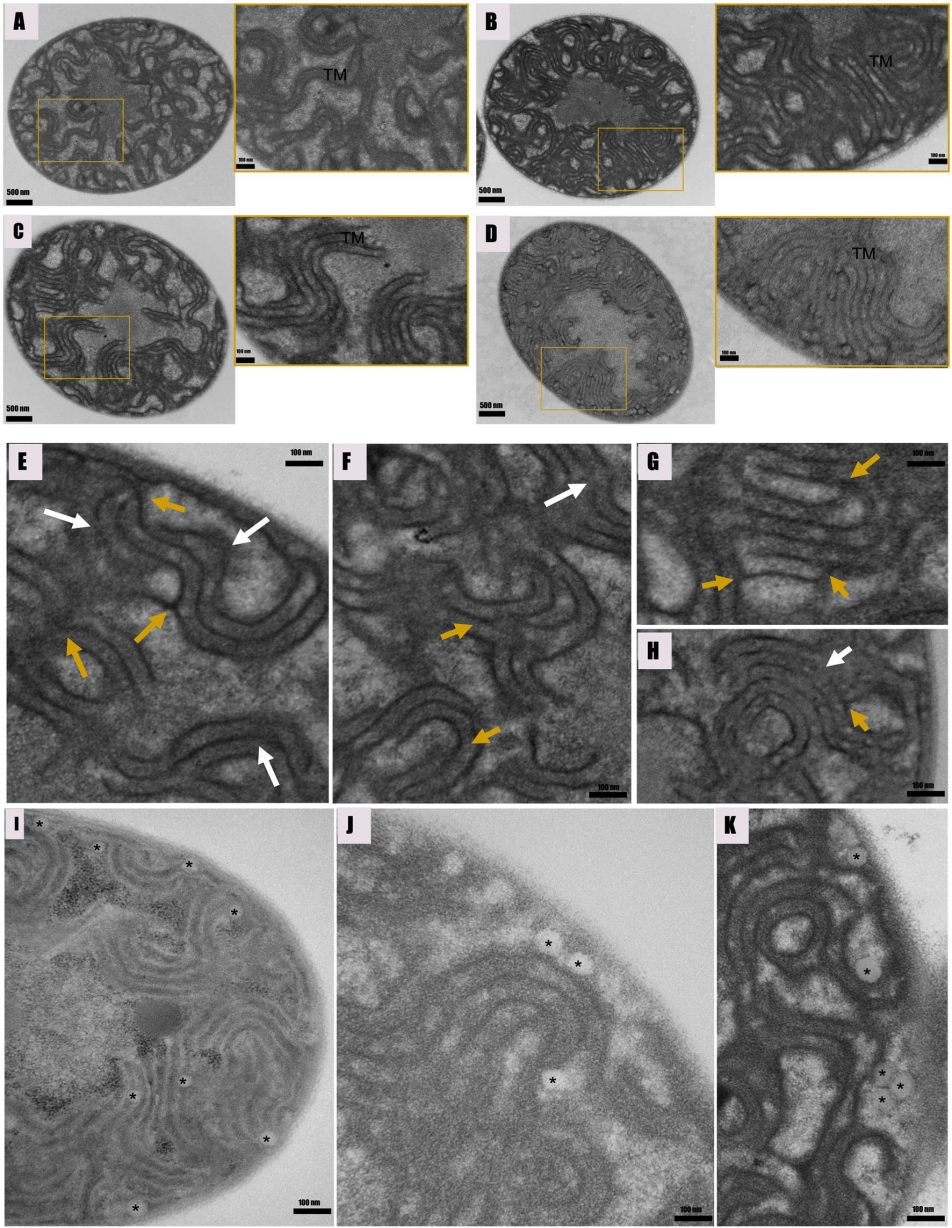
Cross sections of *Fremyella displosiphon* untreated control (**A**), 0.8 mg/L ampicilin (**B**), 3.2 mg/L nZVIs (**C**), and combination of regimen of 0.8 mg/L ampicilllin and 3.2 mg/L nZVIs (**D**) as seen by transmission electron microscopy (TEM). Single layered thylakoid membrane in the untreated control (**A**), complex stacked membranes (5–8 layers) in ampicillin (**B**), nZVIs-treated cells (**C**), and the combinations regimen (**D**–**H**) was observed. The ampicillin and nZVI-treated groups showed siginificant increase in membrane connections that split (yellow arrows) or branch (white arrows). Lipid bodies (asterisks) were dispersed throughout the peripheral ctyoplasm and often juxtaposed with cytoplasmic and thylakoid membranes in ampicillin and nZVI-treated cells (**I**–**K**). TEM images exhibited close associations between these lipid bodies and the thylakoid and cytoplasmic membranes

Distinct differences in the distribution and organization of the thylakoid membrane system in cyanobacterial cells have revealed the evolutionary patterns and structural plasticity of the membrane systems [44]. Despite unveiling the complexity and spatial details of membrane systems, it remains unclear if cyanobacteria can regulate their thylakoid membrane systems in response to environmental stressors such as antibiotic exposure and changes in growth conditions. In this study, we compared the intracellular structural adaptations in nZVI and ampicillin-treated *F. diplosiphon* cells. While thylakoids of algae and higher plants have multilayered structures that can be stacked, compressed, or detached, the thylakoids of cyanobacteria are composed of basic uniform sheets [45]. In our study, we observed single-layered thylakoid membranes in the untreated control. However, complex stacked membranes (5–8 layers) were observed in ampicillin and nZVI-treated cells as shown in Fig. 7C. In accordance, a study by Liberton et al. [46] demonstrated the robust structural flexibility in the architecture of the thylakoid system, resulting in a narrowing of the center-to-center distances in response to light and dark conditions. We observed decrease in the distance between the thylakoid membrane layers on a center-to-center basis in ampicillin and nZVI-treated cells. A previous study has shown that photosynthetic membranes maintain homeostasis by possessing multiple perforations and internal bridges created by branching [46]. We also observed that an increase in the number of thylakoid membrane layers enhanced perforations and fusion sites between them (Fig. 7E–H).

#### Lipid droplets

Cytoplasmic inclusions are located in the central cytoplasmic region with the exception of the lipid bodies, which are found mainly in the periphery [47]. Notably, we observed abundant intracellular inclusions which were peripherally located and presumed to be lipid droplets in antibiotic and nZVI-treated cells (Fig. 7I–K). Additionally, van de Meene et al. [48] reported that lipid bodies were distributed in the peripheral cytoplasm and were often located near the cytoplasmic or thylakoid membranes, suggesting their role in maintaining or generating thylakoids. In addition, Tauchi-Sato et al. [49] reported that a thin, electron-dense layer reminiscent of a phospholipid monolayer binds the lipid bodies as evidence of thylakoid biogenesis. Accordingly, we observed a correlation between increased thylakoid membrane abundance and the number of lipid bodies. Since lipid bodies serve as a source of energy and carbon, an increase in these structures directly correlates to an increase in biofuel production.

## Conclusion

In this study, we report the effect of 0.8 mg/L ampicillin and 3.2 mg/L nZVIs on lipid alterations and cellular effects in *F. diplosiphon* strain B481-SD. Although we observed significant increases in total lipid abundance and FAME composition in the combined treatment of 0.8 mg/L ampicillin and 3.2 mg/L nZVIs, it should be noted that different cyanobacterial strains could exhibit varying levels of tolerance to the combination regimen. Therefore, it is pertinent to calibrate the optimal concentrations required for specific strains/species. Results of the study pave the way to develop cost-effective large-scale lipid synthesis for economical biofuel production. Furthermore, fluorescent-based lipid detection techniques such as Nile red and BODIPY can be combined with solvent-based techniques such as GC × GC/TOF–MS for the assessment of specific lipid groups in cyanobacteria, as well as microscopic analysis using TEM and confocal fluorescence. Future studies will aim at evaluating the combined effect of ampicillin and metallic nanoparticles such as zero-valent iron nanoparticles in facilitating lipid gene transcription activities.

## Data Availability

All the material is owned by the authors and/or no permissions are required.
